# Design and assembly of plant-based COVID-19 candidate vaccines: reсent development and future prospects

**DOI:** 10.18699/VJGB-22-39

**Published:** 2022-05

**Authors:** E.A. Uvarova, P.A. Belavin, E.V. Deineko

**Affiliations:** Institute of Cytology and Genetics of the Siberian Branch of the Russian Academy of Sciences, Novosibirsk, Russia; Institute of Cytology and Genetics of the Siberian Branch of the Russian Academy of Sciences, Novosibirsk, Russia; Institute of Cytology and Genetics of the Siberian Branch of the Russian Academy of Sciences, Novosibirsk, Russia Tomsk State University, Tomsk, Russia

**Keywords:** plant-based vaccines, plant expression systems, virus-like particles, transient expression, stable expression, recombinant proteins, растительные вакцины, системы экспрессии растений, вирусоподобные частицы, транзиентная экспрессия, стабильная экспрессия, рекомбинантные белки

## Abstract

An outbreak of a new variant of the coronavirus infection, known as COVID-19, occurred at the end of 2019 in China, in the city of Wuhan. It was caused by the SARS-CoV-2 virus. This variant of the virus is characterized by a high degree of variability and, as the current situation with its spread across different regions of the globe shows, it can lead to a progressive spread of infection among the human population and become the cause of a pandemic. The world scientific community is making tremendous efforts to develop means of protection,prevention and treatment of this disease based on modern advances in molecular biology, immunology and vaccinology. This review provides information on the current state of research in the field of vaccine development against COVID-19 with an emphasis on the role of plants in solving this complex problem. Although plants have long been used by mankind as sources of various medicinal substances, in a pandemic, plant expression systems become attractive as biofactories or bioreactors for the production of artificially created protein molecules that include protective antigens against viral infection. The design and creation of such artificial molecules underlies the development of recombinant subunit vaccines aimed at a rapid response against the spread of infections with a high degree of variability. The review presents the state of research covering a period of just over two years, i. e. since the emergence of the new outbreak of coronavirus infection. The authors tried to emphasize the importance of rapid response of research groups from various scientific fields towards the use of existing developments to create means of protection against various pathogens. With two plant expression systems – stable and transient – as examples, the development of work on the creation of recombinant subunit vaccines against COVID-19 in various laboratories and commercial companies is shown. The authors emphasize that plant expression systems have promise for the development of not only protective means under conditions of rapid response (subunit vaccines), but also therapeutic agents in the form of monoclonal antibodies against COVID-19 synthesized in plant cells.

## Abstract (ru)

Вспышка нового варианта коронавирусной инфекции, известной как COVID-19, произошла в конце 2019 г. в Китае, в городе Ухань, и была вызвана вирусом SARS-CoV-2. Данный вариант вируса характеризуется высокой степенью изменчивости и, как показывает сложившаяся ситуация с его распространением по различным регионам земного шара, способен приводить к прогрессирующему распространению инфекции среди человеческой популяции и становиться причиной возникновения пандемии. Мировое сообщество исследователей прилагает огромные усилия для разработки средств защиты, профилактики и лечения этого заболевания, основываясь на современных достижениях в области молекулярной биологии, иммунологии и вакцинологии. В предлагаемом обзоре суммирована информация о современном состоянии исследований в области создания вакцин против COVID-19 с акцентом на роль растений в решении этой сложной проблемы. Хотя растения издавна использовались человечеством в качестве источников различных лекарственных субстанций, в условиях пандемии растительные системы экспрессии становятся привлекательными в качестве биофабрик или биореакторов для наработки искусственно созданных белковых молекул, включающих протективные антигены для вакцины против вирусной инфекции. Дизайн и конструирование таких искусственных молекул лежат в основе создания рекомбинантных субъединичных вакцин, нацеленных на быстрое реагирование против распространения инфекций с высокой степенью изменчивости. В обзоре представлено состояние исследований, охватывающее период немногим более двух лет, т. е. с момента появления новой коронавирусной инфекции. Обсуждается важность быстрого реагирования исследовательских групп из разных научных областей в направлении использования уже существующих разработок для создания средств защиты против различных патогенов. На примере двух растительных систем экспрессии – стабильной и транзиентной – показано развитие работ по конструированию рекомбинантных субъединичных вакцин против COVID-19 в различных лабораториях и коммерческих компаниях. Подчеркнута перспективность использования растительных систем экспрессии для разработки не только средств защиты в условиях быстрого реагирования (субъединичные вакцины), но и терапевтических средств в виде моноклональных антител против COVID-19, синтезируемых в растительных клетках.

## Introduction

Vaccination is one of the most effective methods of combating infectious diseases. A vaccine is a preparation that stimulates the body to form a protective reaction against an infectious agent. Vaccination is based on the programming of specific immunological mechanisms for protection against pathogens of various infections. Although humanity has managed to avoid outbreaks of many dangerous infections precisely thanks to vaccination, the vaccines available in the arsenal are still far from “ideal”. The use of traditional vaccines, the production of which is based on attenuated or inactivated pathogens, is sometimes accompanied by sensitization of the body, a large load on the immune system, reactogenicity, toxicity, etc. (Francis, 2018).

The methods and approaches developed to date in the field of molecular biology, immunology, vaccinology, cellular and synthetic biology, as well as bioinformatics, allow us to take a fresh look at the opening opportunities for creating more advanced means of protection against pathogens of viral and bacterial origin, devoid of the above disadvantages. The use of modern biology methods makes it possible to identify and isolate biological macromolecules or their fragments that could be used as immunogenic components to activate the immune system in response to a pathogen. Such components can be proteins of pathogens (for example, envelope proteins of infectious agents), which are immunogens. With the use of genetic engineering technologies, the direction of creating recombinant subunit vaccines is successfully developing – artificially created protein molecules that include protective antigens in combination with adjuvants synthesized in various expression systems (Salazar-González et al., 2015; Demurtas et al., 2016; Fischer, Buyel, 2020; McNulty et al., 2020; Rybicki, 2020)

The creation of recombinant subunit vaccines is most relevant for pathogens characterized by a high level of variability. These pathogens include viral pathogens that cause acute respiratory infections and influenza (Shoji et al., 2011; Ward et al., 2020). These pathogens can lead to a progressive spread of infection in the human population and cause epidemics and pandemics. These pathogens include a new type of coronavirus – SARS-CoV-2.

The availability of data on the genome structure of a new virus strain isolated using classical methods of virology, electron microscopy, and molecular analysis at the end of 2019 (Zhu et al., 2020) opened up wide opportunities for applying new approaches to designing vaccines. The first statements about clinical trials (https://clinicaltrials.gov/ct2/show/ NCT04283461) appeared two months after the publication of the primary structure of the genome of this virus (Zhu et al., 2020). This fact indicates that the existing developments and understanding of the molecular mechanisms of the formation of protective reactions on the part of the body’s immune system make it possible to respond quickly enough to the emergence of new variants of a viral infection (Pogrebnyak et al., 2005). However, the use of new vaccines for the prevention of the population is dictated by the need for a deep assessment of their effectiveness and impact on the human body, as well as the possibilities for their industrial development (Jiang, 2020).

In this review, the authors made an attempt to analyze the possibilities of using plant expression systems aimed at creating antiviral subunit recombinant vaccines, in particular, candidate vaccines against COVID-19.

## Plant-based expression systems

When developing new generation vaccines, including recombinant vaccines, the question of finding highly effective and cost-effective systems for their expression remains topical. Currently, Escherichia coli, several species of Saccharomyces, and mammalian cells are most commonly used for these purposes. New prospects for the production of recombinant proteins are opening up with the use of recombinant plants (biopharming) that could act as plant (edible) vaccines (Salazar-González et al., 2015). Plants in whose tissues recombinant immunogens are synthesized and accumulated are attractive for obtaining substances for veterinary and medical purposes, including for obtaining subunit recombinant antiviral vaccines. Many leading biotechnology laboratories and commercial firms use plant cells as an alternative expression system for the production of recombinant proteins for medical purposes (Fischer, Buyel, 2020; Rybicki, 2020).

**Fig. 1.**
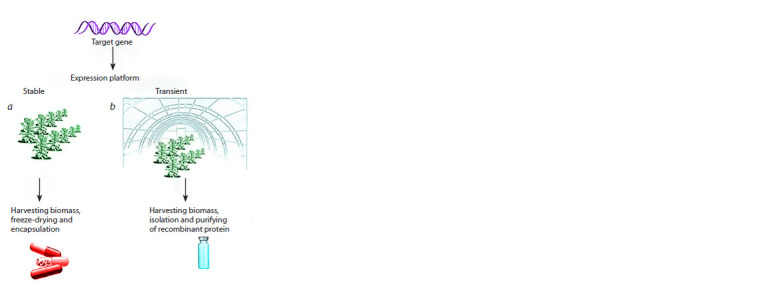
Plant-made expression platforms used for the synthesis of recombinant proteins

Plant-made expression platforms used for the synthesis of recombinant proteins, including vaccinogenic ones, are based on stable expression of the target gene when it is delivered to the nuclear or chloroplast genomes of the plant, as well as on its transient expression. Figure 1 shows two main platforms being developed in leading biotechnological centers for the production of recombinant proteins, including medical ones, using the synthetic capabilities of the transcription-translation apparatus of plants. The general principle underlying these platforms is as follows: by genetic engineering, an artificial matrix with a target gene is created, according to which the corresponding protein is synthesized and accumulated in plant tissues. Plant tissues can be freeze-dried and encapsulated, or the recombinant protein can be isolated and purified directly from the tissues. As a rule, genes of envelope proteins of pathogens of infectious diseases, which are immunogens, are used as a target gene. As part of expression cassettes, target genes can be integrated into the plant genome (nuclear or chloroplast), which will ensure stable expression of the target gene and accumulation of the target product in plant tissues (see Fig. 1, a). However, the use of the chloroplast genome for these purposes, although it seems very promising, is still far from practical application due to the existence of a large number of unsolved problems (Waheed et al., 2015; Yu Y. et al., 2020).

To deliver target genes to plant tissues in a transient expression system, viral vectors specifically designed for this purpose are used (Sainsbury et al., 2010), as well as the Nicotiana benthamiana plant species, the structural features of the leaf parenchyma of which are optimal for successful agroinfiltration (see Fig. 1, b). IconGenetics (Germany) has developed and patented a “magnification” system in which the yield of a recombinant protein in a transient expression system can reach up to 80 % of the total soluble protein (TSP) (Gleba et al., 2005). Despite the relatively low (slightly more than 1 % ORP) yield of the recombinant protein in plants with a stable expression system in the case of nuclear transformation, the use of already available agricultural technologies for growing transgenic plants provides them with unlimited scalability at minimal cost (Kermode, 2018). Thus, a plant platform with stable expression of the target gene is promising for the production of high-volume products, such as vaccines for disease prevention, especially in developing countries

Transgenic or transplastomic plants with stable expression of the target gene are used for large-scale production of recombinant proteins over a long period, while the characteristics of transient expression make it possible to obtain the required amounts of recombinant protein in short periods of time, which seems to be extremely important when an emergency response to the spread of a pathogen is required. For example, the US Food and Drug Administration (FDA) approved an emergency cocktail against Ebola virus called ZMappTM, consisting of three monoclonal antibodies transiently synthesized in tobacco plants (Phoolcharoen et al., 2011).

The transient expression system is promising for the development of small-scale accumulation of personalized drugs, such as anti-idiotypic scFv antibodies for non-Hodgkin’s lymphoma, as well as in case of a need for mass vaccination of the population in case of outbreaks of seasonal viral diseases caused by rapidly mutating viruses. In N. benthamiana plants, after three weeks from the moment the viral sequence was already isolated, sufficiently large amounts of antigens were synthesized from influenza virus strains H5N1 (bird flu) and H1N1 (swine flu) (Hodgins et al., 2019; Makarkov et al., 2019). These recombinant proteins, synthesized in a transient plant expression system, are being considered as candidate influenza vaccines and have completed phase II human trials (Pillet et al., 2019).

DowAgroSciences (USA) has developed the Concert™ plant cell culture system as an advanced platform for the production of a recombinant antigen against Newcastle disease virus (pseudoplague) in poultry. Although the company did not launch commercial production of this recombinant vaccine, this technology has served as the basis for other commercial products. The reality and effectiveness of this approach has been repeatedly confirmed by researchers from the world’s leading biotechnological laboratories, as well as by the activities of numerous companies and firms specializing in the production of one or more closely related products based on their own expression platform (Margolin et al., 2018; Rybicki, 2018).

Given the dramatic impact of the COVID-19 pandemic, it is critical to consider all the technologies at the disposal of researchers that could be applied to combat the causative agent of this infectious disease, the SARS-CoV-2 virus. Since the technology for the production of plant biopharmaceuticals has already been generally developed, it seems very attractive in the context of a pandemic in terms of producing not only inexpensive vaccines, but also antibodies used for therapy, prevention and diagnosis. The production of antibodies, such as anti-COVID-19, seems even more promising than vaccinogenic proteins, since recombinant plant-derived antibodies can be produced and approved for human use in a timely manner compared to vaccine development (Hiatt et al., 1989; Tian et al., 2020). The promise of plant expression systems for use in the fight against COVID-19 is discussed in reviews (Rosales- Mendoza, 2020; Shanmugaraj et al., 2020b).

## General idea of the immune response to viral infection

The causative agents of respiratory diseases, which include various types of coronaviruses, enter the human body through the mucous membranes of the upper respiratory tract. Virus particles attach to cell receptors, fuse with the cell membrane, and enter the cell. Using the replicative apparatus of the cell, the virus multiplies and the viral particles come out, affecting the cells adjacent to it. In the case of the SARS-CoV-2 virus, penetration into the cell is provided by the S-2 protein, which is one of the two parts of the surface viral S protein (spike protein). The second part of this protein – S-1 provides binding to the ACE2 receptor of the lung epithelium. Having penetrated inside the cell, viruses become intracellular parasites, and the fight against them by the host’s immune system becomes a difficult task.

**Fig. 2.**
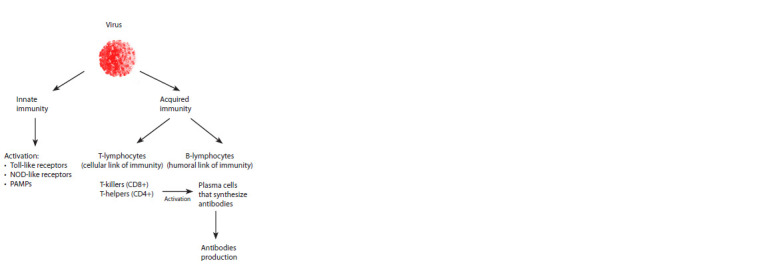
Scheme of the immune response of the body to the invasion of a viral infection

Evolutionary, two systems of protecting the body from the penetration of pathogens have been formed – innate, immediately responding to danger, aimed at identifying the pathogen as a whole (innate immunity) and adaptive, aimed at identifying a huge number of specificities (antigens) in various pathogens (acquired immunity). The molecular mechanism of pathogen recognition is based on the detection of some standard “molecular marks” or pathogen-associated molecular patterns (PAMPs). Figure 2 shows the general scheme of the development of the body’s immune responses to the penetration of the virus.

Innate immune responses are triggered at the first stage of interaction between the organism and the pathogen. Pathogen structures are recognized by the receptors of phagocytic cells and natural killers, upon interaction with which T-cell immune response cascades are launched and the elimination of pathogens and infected cells is coordinated. Toll-like and NOD-like receptors constitute the main group of receptors during the development of nonspecific protection (Takeuchi, Akira, 2010; Channappanavar et al., 2014).

It should be emphasized that in defending itself against viruses, the cell uses both antibodies (the humoral link of immunity) and the strategy of destroying cells infected with the virus (the cellular link of immunity). Membrane proteins of most viruses are “identification marks” or targets for the cell, on which B-lymphocytes activated by T-helpers (CD4+) differentiate into plasma cells that synthesize antibodies (see Fig. 2), which prevent the attachment and penetration of the virus into the cell. Such a defense strategy is effective in the early stages of infection, until the virus has entered the cell. After a cell is infected with a virus, another strategy is activated to destroy them, which is carried out by natural killers and cytotoxic T-lymphocytes (CD8+) (see Fig. 2). The importance of the formation of cytotoxic reactions in the fight against coronaviruses was emphasized earlier (Channappanavar et al., 2014). The scheme of immune responses of the body to the invasion of a viral infection, shown in Figure 2, is extremely simplified in order to draw attention to the key points that are important when choosing a strategy for developing a vaccine.

## Principles for COVID-19 vaccine development

Modern knowledge in the field of molecular biology, immunology and vaccinology provides researchers with a wide range of methods and approaches for designing new generation vaccines based not only on data on the antigenic structure of the pathogen, but also on the mechanisms of the immune response to the pathogen and its components

Nucleotide sequences of the SARS-CoV-2 virus genome are available on the websites of the National Library for Medicine and the Gene Bank (https://www.ncbi.nlm.nih.gov/ sars-cov-2/). As of mid-July 2021, information on more than 377,000 fully read genomes of this virus, as well as more than 526,000 partially read genomes, is freely available. Since the outbreak of a new coronavirus infection COVID-19 occurred at the end of 2019 in China, in the city of Wuhan, the nucleotide sequence of this strain of the virus was conditionally chosen as a reference. Reference sequence data and all sequenced genomes are available from the gene bank at https:// www.ncbi.nlm.nih.gov/nuccore/NC_045512.2.

At this point in time, on the WHO website, you can find information on the status of completed developments for the creation of vaccines against SARS-CoV-2 (https://cdn.who.int/media/ docs/default-source/blue-print/15april2022-novel-covid-19- vaccine-tracker.zip?sfvrsn=225505e5_3&download=true). It should be emphasized that to date, 196 candidate vaccines at the stage of preclinical studies and 153 preparations at the stage of clinical trials have been registered in the world. On the WHO website via the same link, you can also find information about specific vaccine preparations and their manufacturers that are at the stage of clinical trials and receiving approval as vaccines from WHO (see the Table).

**Table 1.**
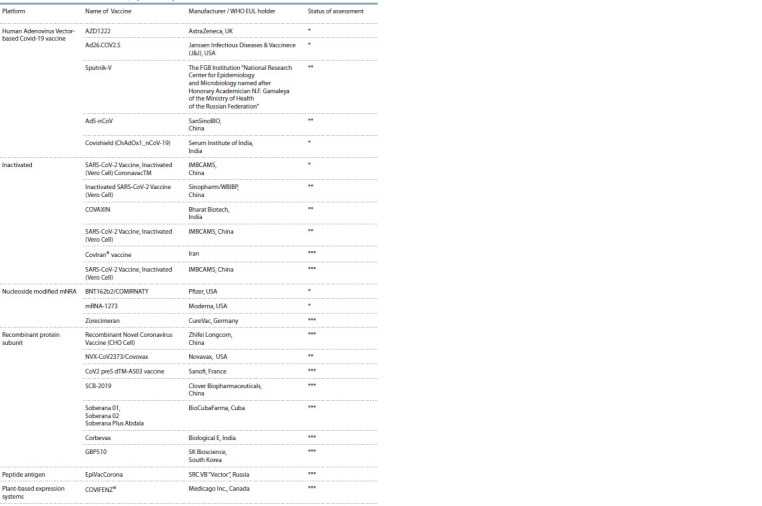
Status of COVID-19 vaccines within WHO EUL/PQ evaluation process 02.04.2022 Note. WHO website was used: https://extranet.who.int/pqweb/sites/default/files/documents/Status_COVID_VAX_02April2022.pdf Status of assessment: * registered, ** end of registration, *** preparatory procedures.

Analyzing the state of research in the development of vaccines against COVID-19, it should be noted that almost all creators use the immunogenic protein S of the coronavirus as a basis, which is presented to the immune system in different ways. It is this protein of the SARS-CoV-2 virus that binds to the ACE2 receptor of mucosal epithelial cells and ensures its penetration into the cells of the human body. As can be seen from the results of the analysis of the status of vaccines that have been registered and prepared by developers for use (see the Table), the current market for COVID-19 vaccines includes both classical vaccines based on the presentation of antigens of inactivated viruses to the immune system (Gao et al., 2020) and mRNA vaccines, in which the mRNA encoding the S protein is packaged in a lipid envelope. Such mRNA, when it enters human cells, is a template for the synthesis of the S protein, which is recognized by the cells of the immune system as a danger signal (Pardi et al., 2018). RNA vaccines have been shown to induce neutralizing antibodies with high titers (Jackson et al., 2020). Based on the same mechanism of antigen presentation, DNA vaccines are being developed that include a DNA fragment encoding the S protein into vectors, for example, into plasmids or adenoviruses (see the Table). In studies in rhesus monkeys, such vaccines stimulated the production of high antibody titers as well as the production of cytotoxic lymphocytes (Yu J. et al., 2020). The disadvantage of vector vaccines is the immunogenicity of the vectors themselves.

Vaccines based on recombinant proteins or peptides are considered promising. In the case of SARS-CoV-2, full-length or domains of S, M, and N proteins are considered as candidates for antigens, to increase the immunogenicity of which epitopes recognized by T- and B-cells of the immune system are additionally used (Marian, 2021). Such artificially created recombinant proteins, when they enter the body, activate the cells of the immune defense systems, which trigger the formation of the corresponding subcellular populations, the biosynthesis of antibodies, and the formation of “memory cells”. The most complete strategy for creating vaccines against SARS-CoV-2 and the current state of research in this area are presented in the review (Bakhiet, Taurin, 2021).

It should be emphasized that in the development of antiviral vaccines, including against COVID-19, two important stages can be conditionally distinguished, the first of which is directly related to the creation of the vaccine itself, which is presented to the immune system either in the form of a large number of antigens (inactivated virus), or in the form of the dominant antigen(s) in the form of mRNA, DNA, recombinant protein or peptide. The importance of the second stage is determined by reliable systems for the production of either the virus itself or its antigens. Analyzing the current state of research in the development of vaccines and, in particular, subunit vaccines of a new generation, it should be noted that, along with wellestablished platforms, for example, Chinese hamster cells (CHO), used in the development of the recombinant vaccine ‘Recombinant Novel Coronavirus Vaccine’ by the Chinese company Zhifei Longcom (see the Table), plant expression systems are attracting increasing attention of the global research community (Fischer, Buyel, 2020; Kannan et al., 2020).

## The state of research in the development of plant-based vaccines against COVID-19

Despite the fact that the first work on the attractiveness of plant expression systems for the biotechnological production of vaccinogenic proteins against COVID-19 appeared relatively recently (Rosales-Mendoza, 2020; Shanmugaraj et al., 2020a), by now the number of such works has increased markedly (Capell et al., 2020; Dhama et al., 2020; Ma et al., 2020; Prasad et al., 2020; Shanmugaraj et al., 2020a). The authors comprehensively discuss the possibilities of applying existing biotechnological developments to create subunit vaccines based on plant expression systems (Capell et al., 2020; Ma et al., 2020), as well as the features of creating this type of vaccine in case of a need for rapid response to the spread of a pathogen (Shanmugaraj et al., 2020a).

The researchers’ developments on the creation of plantbased influenza vaccines based on virus-like particles also formed the basis for the development of vaccines against COVID-19 (Hodgins et al., 2019; Makarkov et al., 2019).

Moreover, the Canadian company ‘Medicago’, which uses plant expression systems for the production of recombinant proteins for medical purposes, used a transient expression system in N. benthamiana plants to develop a vaccinogenic protein including S-1 protein of the SARS-CoV-2 virus (https://www.medicago.com/en/covid-19-programs/). The company’s developers used the fusion of a sequence encoding the viral protein S-1 with a sequence providing conformational transformations of a protein molecule that mimic the surface of a viral particle. The prospects of creating recombinant proteins conformationally folded in the form of virus-like particles, on the surface of which antigens are presented in the form of recombinant polypeptides, were noted earlier (Bai et al., 2008). Folding a recombinant protein in the form of a virus-like particle significantly increases the efficiency of antigen presentation to cells of the body’s immune system (Rybicki, 2020). Despite the fact that the vaccine prepared on the basis of virus-like particles, although it mimics a virus, such an “artificial virus” lacks a genetic apparatus (RNA or DNA) and, accordingly, the ability to replicate when it enters a cell. Previously, numerous studies have shown that post-translational protein transformations in plant expression systems ensure its folding into a virus-like particle (D’Aoust et al., 2010; Lua et al., 2014). Moreover, it has been demonstrated that plant expression systems support the synthesis of functional recombinant proteins, including such complex ones as antibodies (Diamos et al., 2020).

The website of the biotech company Medicago Inc. published information on the approval by the Canadian regulator of a vaccine, which is a recombinant S protein of the SARS-CoV-2 virus, in the form of virus-like particles synthesized in tobacco plants (N. benthamiana) (https:// medicago.com/app/uploads/2022/02 /Covifenz-PM-en.pdf). The vaccinogenic protein isolated and purified from plant biomass is used in tests on volunteers (about 30 thousand people participated in the experiment). It should be emphasized that the candidate vaccine obtained on the basis of the plant expression system has successfully passed three phases of clinical trials on volunteers (Pillet et al., 2019; Ward et al., 2021) and is now approved under the commercial name COVIFENZ® in Canada. The company announced the formation of high antibody titers in the subjects. Medicago Inc. has evaluated its candidate vaccine with GSK pandemic adjuvant. Currently, companies such as GlaxoSmithKlein (GSK, UK), Seqirus (UK) and Dynavax (USA) are developing licensed adjuvants (AS03, MF59 and CpG 1018, respectively) for their use with COVID-19 vaccines. The use of an adjuvant may be of particular importance in a pandemic situation, as it can reduce the amount of vaccine protein required per dose, which allows more doses of the vaccine to be produced and therefore contributes to the protection of more people. Although the exact dosage of the vaccine for humans has not yet been determined, the company estimates potential production volumes starting with 2021 to 80 million doses per year with an increase in productivity from 2023 to more than 1 billion doses of the COVID-19 vaccine per year

## Conclusion

As the experience of leading biotechnology companies and laboratories in optimizing expression systems for the production of recombinant proteins shows, plant expression systems are very attractive for these purposes and are already in demand by some large and medium-sized biotech companies. The prospect of using plant cells for the production of recombinant proteins intended for vaccine prophylaxis is also based on the possibility of their oral and intranasal administration and the activation of mucosal responses.

Oral delivery of pharmaceutical proteins appears to be a desirable target for the biopharmaceutical industry, as it provides more convenient drug administration than intravenous, intramuscular, and subcutaneous injections. Oral delivery will lead to better patient outcomes along with improved quality of life. Moreover, the attractiveness of plant expression systems is based on the ability to quickly respond to pathogens with a high degree of variability. The examples of successful testing of a plant vaccine against COVID-19 and the provision of large production volumes for the production of a vaccinogenic recombinant protein given in this review confirm the promise of plant expression systems for obtaining recombinant subunit vaccines

## Conflict of interest

The authors declare no conflict of interest.
